# Failure to Control Caries in an AIDS-Affected Individual: A Case Report

**DOI:** 10.1155/2012/643436

**Published:** 2012-02-12

**Authors:** Karen A. Nedwick-Castro, Alexandre R. Vieira

**Affiliations:** ^1^Department of Operative Dentistry/Comprehensive Care, School of Dental Medicine, University of Pittsburgh, 3501 Terrace Street, 3074 Salk Annex, Pittsburgh, PA 15261, USA; ^2^Department of Oral Biology, School of Dental Medicine, University of Pittsburgh, 3501 Terrace Street, 614 Salk Hall, Pittsburgh, PA 15261, USA

## Abstract

Many patients today are living longer with certain health issues like human immunodeficiency virus (HIV). These patients take numerous medications (HAART: highly active antiretroviral therapy) that cause xerostomia, which increases caries risk. There are particular challenges when treating HIV-positive patients with decreased immune systems, which can also accelerate the progression of periodontal disease. This paper discusses one such case that was followed from 2002 to 2010 at the University of Pittsburgh School of Dental Medicine, where an HIV patient lost all his teeth despite investing thousands of dollars. It is also common sense that just performing restorations to decayed teeth is not enough to control the disease, but recommending a personalized preventive maintenance program to the patient is a must.

## 1. Introduction

Dental practitioners are having some of the same issues with treating caries that practitioners had decades ago. Despite all of our knowledge about the pathogenesis of the disease (plaque removal, prescription fluorides, and discussion about high carbohydrate diets), in some patients we never really get control of their disease. The patient comes in for recall examinations only to find out they either have new/recurrent carious lesions. There comes a point in time that we cannot keep restoring teeth and many may become nonrestorable, which leads to frustration for the practitioner and patient. In the case presented here, the patient had many risk factors for oral disease such as HIV, smoking, poor oral hygiene, irregular cleanings, a high-carbohydrate diet, and xerostomia. As important as it is to restore the lesions, the practitioner has to enlist the patient in his own home care and educate the patient on their own unique risk factors and how to eliminate them for there to be successful treatment outcomes. 

## 2. Case Report

A 38-year-old Caucasian male came to the University of Pittsburgh School of Dental Medicine for a comprehensive exam on October 8, 2002. His primary reason for seeking dental care was that he thought he may have cavities after a jaw fracture approximately one year prior. He was in a car accident in which he not only broke his jaw, but his leg, shoulder, wrist, and arm. He wanted to get as much dental work done as possible, because he now had dental insurance.

The patient's medical history was positive for HIV infection, of which he had for 14 years and was well controlled. He was taking numerous medications, two of which were antiretroviral agents for HIV: Didanosine (Videx) and Tenofovir (Viread). Didanosine is known to cause xerostomia. He was also taking the antidepressant Sertraline (Zoloft) known also to cause xerostomia. The patient did not report any discomfort related to dry mouth at this time. The remaining medications were Atorvastatin (Lipitor), Ranitidine (Zantac), Loperamide (Imodium), and Triazolam. The patient was allergic to sulfa drugs. He also smoked one and a half pack of cigarettes per day for the last 20 years. 

**Figure 1:**
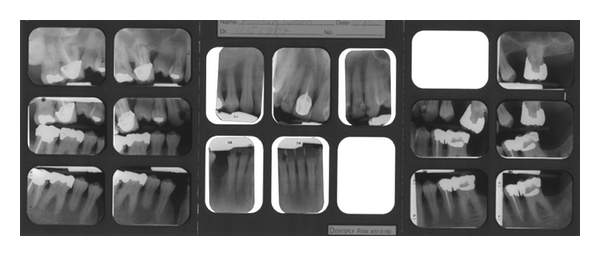
Full-mouth series 11-08-2002.

Upon clinical examination, the patient presented with rampant caries and moderate periodontitis. It was recommended a therapy consisting of scaling and root planning in all four quadrants (with three-month recalls) and restoring 14 carious lesions. Once the disease control phase would be completed, we could move on to definitive treatment. The patient came in routinely for his dental appointments, and all disease control procedures were completed by December 11, 2002 (Figure 1). 

The patient presented approximately nine months later for a recall examination. The patient had no major changes in his medical history other than his medications. He is now taking four medications for HIV: Saquinavir (Invirase), Lopinavir/Ritonavir (Kaletra), Didanosine (Videx), and Tenofovir (Viread). Other medications included Atorvastatin (Lipitor), Ranitidine (Zantac), Mirtazapine (Remeron), and Morphine. At that time, periodontal and prosthodontic evaluations were completed and it was recommended to extract teeth units 3, 4, and 14 due to advanced periodontal disease and a failed root canal and the execution of a maxillary removable partial denture (RPD). It was also recommended that the patient replace a crown on tooth unit 31 due to recurrent caries/defective margins; he preferred it to be patched but never came back to have it done. It was also recommended that the patient have a crown on tooth unit 20 due to a root canal and large restoration; he, however, did not want it completed at this time. He had two new carious lesions that were restored on the maxillary and a prophylaxis was completed. The final maxillary RPD was inserted December 23, 2003. 

**Figure 2:**
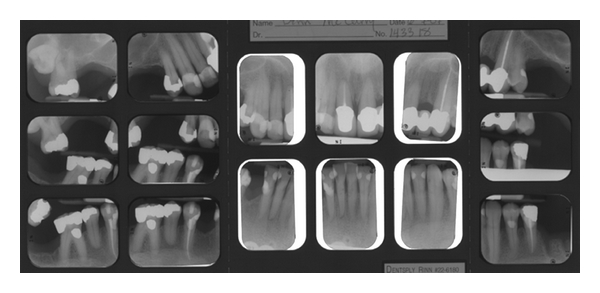
Full-mouth series 06-07-07.

**Figure 3:**
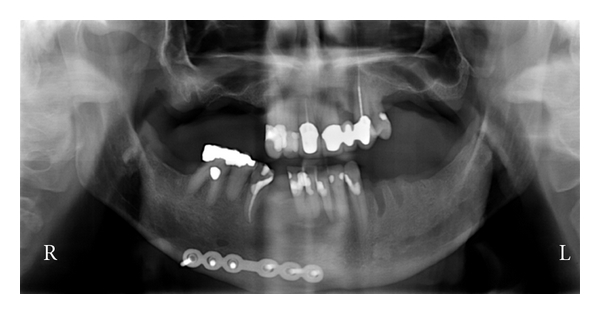
PAN 07-17-09.

On August 3, 2004, the patient presented for a recall examination only to find nine carious lesions (some recurrent caries under existing crowns) and the progression of his periodontal disease. At this time, xerostomia was noted and his smoking remained consistent. Due to the fast progression of caries and periodontal deterioration, an option of full mouth extractions with complete maxillary and mandibular dentures were discussed. The patient after careful consideration still wanted to save his teeth with crowns, root canals, restorations, and regular cleanings. It was discussed that due to the progression of dental disease that he had a fair to questionable prognosis for treatment. In the next months, the patient came in for his cleaning and restorative work. During the treatment, he was due for another recall examination (medications the same and CD4 count was 735) in which scaling and root planning was recommended again. During one of the visits, the carious lesion on one tooth was so extensive that a root canal was completed, but there was subsequent fracture and the tooth had to be extracted and an anterior three-unit bridge (from unit 9 to 11) was recommended, as well as replacing a single crown on tooth unit 8. This event forced the treating doctor into definitive work to reestablish aesthetics without ever having control of the oral disease process. The bridge and single crown were completed along with other work on May 9, 2006. The patient continues to come to the clinics for another recall examination on August 10, 2006; his health history was unchanged, with one new medication [Duloxetine (Cymbalta)], and CD4 count of 550. At that time, more scaling and root planning was recommended with some restorative and an extraction of a posterior tooth (Figure 2). The patient begins to get frustrated in the remaining months/years; he does not come in for all the recommended treatment and now only shows up for emergencies (a total of five emergency visits); he also notices that the crown on tooth unit 8 is feeling loose, during that time he is also having more health issues due to the AIDS. Medications include Saquinavir (Invirase), Tenofovir (Viread), Lopinavir/Ritonavir (Kaletra), Didanosine (Videx), Morphine, Ranitidine (Zantac), Trazodone, Clonazepam, Gemfibrozil, and Dronabinol (Marinol). His CD4 count is 450 now. He finally submits to the idea of full mouth extractions with complete dentures due to his deteriorating oral condition on July 21, 2009. The extractions and fabrication of maxillary and mandibular complete dentures were completed on April 19, 2010 (Figure 3).

## 3. Discussion

There is great interest in providing timely and appropriate treatment to caries using current and emerging evidence regarding caries activity and control. The goal is to plan treatment for and with our patients properly, staging lesion severity, assessing lesion activity, and taking the patient's overall caries risk into account [1]. In looking at this case, the patient had many risk factors for caries: smoking, HIV infection, xerostomia, high carbohydrate diet, and unsatisfactory oral hygiene with irregular frequency of dental cleanings (maintenance). However, proposed treatments had the ability to restore dental function and aesthetics but alone they did not control the patient's caries disease. The patient never considered smoking cessation, which is also known to compromise healing and promote the destruction of the periodontium. It would have been important to reinforce at each visit the need for highly efficient home care. Higher HIV viral loads associate with greater levels of periodontal destruction and more severe caries [2]. AIDS has also been shown to be associated with salivary gland hypofunction and/or xerostomia [3].

The patient presented here was taking many medications (HAART) throughout treatment that were known to cause decreased salivary flow [3], and there is evidence that HAART is associated with increased caries experience [4]. This patient was using breath mints and hard candies when his mouth was dry instead of using water or a saliva substitute like Biotene. He was also snacking frequently between meals, which further promoted caries. However, half way through his treatment he was given a prescription fluoride like PreviDent 5000, which is a prescription toothpaste to be used daily that can deliver 5000 ppm fluoride. Although the prescription was given later in treatment, it was not known if the patient was committed to using it. 

A personalized treatment for the case presented here may had had the different outcome of more longevity of the treatments provided and could have included aggressive fluoride exposure, nonsugar saliva substitutes, and more frequent recalls for oral hygiene monitoring. As early as at the first recall appointment (2004), it became clear that we did not have control of the oral disease. The patient had more carious lesions and the periodontal disease was progressing. The dental practitioners strongly recommended full-mouth extractions with complete dentures. If there is a fair/questionable prognosis in saving the patient's teeth, do you still proceed with treatment? Could we have spared the patient of time, money, and frustration? It is important to identify the signs that may increase the difficulty in controlling caries disease early so costly and inefficient treatment is not proposed before the disease is under control. Personalized treatments are recommended, even if they include a more frequent recall schedule.

## 4. Conclusion

In summary, treating caries disease properly has moved beyond the instant “fill” or “no-fill” call [1]. Cases such as the one presented in this paper require customized treatment that may include more frequent recalls and staged treatments. Dentistry as a profession needs to continue the discussion for the proper compensation of dentists that provide nonoperative/preventive caries management strategies, moving away from a restorative-only strategy, which we know fails in a case such as the one we presented here.
